# Recent Advances in Hydrogels for Tissue Engineering Applications

**DOI:** 10.3390/gels12060454

**Published:** 2026-05-22

**Authors:** Olga Kammona, Evgenia Tsanaktsidou

**Affiliations:** Chemical Process and Energy Resources Institute, Centre for Research and Technology Hellas, P.O. Box 60361, 57001 Thessaloniki, Greece; jtsanaktsidou@certh.gr

Hydrogels are three-dimensional (3D) hydrophilic polymer networks characterized by increased water content (>90%) that have arisen as extremely versatile biomaterials for tissue engineering (TE) applications (e.g., cartilage and bone regeneration, neural tissue regeneration, cardiac repair, corneal tissue repair, wound healing, etc.) (Figure 1) with key roles as scaffolds, dressings, patches and adhesives [1,2]. Their application in TE is mainly due to their biocompatibility/biodegradability, porosity, moisture retention, tunable viscoelasticity, and mimicry of extracellular matrix (ECM), which allow them to provide the necessary biochemical and structural support for tissue regeneration. Furthermore, hydrogels can be easily functionalized to exhibit immunomodulatory and antimicrobial properties as well as responsiveness to various external stimuli such as light, pH, temperature and electric/magnetic fields [1].

Both naturally derived (e.g., hyaluronic acid, collagen, chitosan, gelatin, alginate, etc.) and synthetic (e.g., polylactic acid, polyethylene glycol, etc.) polymers have been utilized for hydrogel formation, with a slight preference towards natural polymers, which exhibit biocompatibility, increased cell attachment and antibacterial activity. It should be noted that the physicochemical, rheological and biological properties of hydrogels can be readily influenced by their chemical composition (i.e., selection of polymer backbones and functional groups, crosslinking reaction, etc.), structural design, and biofunctionalization [1,3]. By precisely tailoring the above, hydrogels can meet the specific regenerative requirements of the different tissues [1]. In this respect, radical hydrogel design has led to the development of smart hydrogels exhibiting multi-stimulus responsiveness, self-healing, and programmable degradation [4].

Hydrogel crosslinking can be physical or chemical. Physical crosslinking refers to noncovalent interactions (e.g., ionic and hydrogen bonds, polymer entanglements) and chemical crosslinking (e.g., free radical polymerization, click chemistry, enzyme-induced crosslinking) refers to covalent bonding and provides higher mechanical stability. Among chemical crosslinking methods, click chemistry (e.g., thiolene reactions, Diels–Alder cycloadditions, tetrazine–norbornene couplings and azide–alkyne cycloadditions) has been recently recognized as a great and flexible strategy for the design/development of injectable, stimulus-responsive, biodegradable and/or multifunctional hydrogels used in TE applications (e.g., cartilage, skin, neural, cardiac, etc.) owing to its high specificity, high reaction rates, and biocompatibility [5].

This Special Issue presents a collection of four original research papers and two review papers focusing on the diverse applications of hydrogels in tissue engineering, by research groups coming from different countries across Europe, America and Asia. The four original research papers cover various topics including (i) hydrogel-aided wound healing via the development of biomimetic cell-laden hydrogels for the treatment of full-thickness skin wounds, and collagen hydrogels loaded with fermented extracts of *Flourensia cernua* (*F. cernua*) for the sustained release of the extracts to the wound environment; (ii) hydrogels for 3D cell cultures such as Thiol-Clickable GelAGE Hydrogels; and (iii) thermoresponsive hydrogels for injectable drug delivery applications such as hydrogels of poly(lactic acid) (PLA) and poly(ethylene glycol) (PEG). The two review papers, presented after the original research papers, cover the state-of-the-art research studies related to (i) the application of hyaluronic acid (HA)-based hydrogels in periodontal treatment via modulation of inflammation and microbial burden, and tissue regeneration, and (ii) the use of hydrogels in cardiac surgery as versatile platforms for tissue repair, adhesion prevention, and localized delivery of therapeutics. A summary of each of the papers published in this Special Issue is presented below.

Healing of full-thickness wounds and regeneration of skin without scarring constitute important challenges in skin tissue engineering. In this respect, the first research paper deals with the development of a multilayer composite biomimetic scaffold (bionic skin) comprising a decellularized amniotic membrane matrix (AM) seeded with epidermal stem cells (EpiSCs) and combined with a fibroblast-laden collagen gel (FCG) to achieve hierarchical regeneration of full-thickness skin wounds. It was shown that the treatment of injured skin with the developed scaffold resulted in rapid skin healing without scarring, regeneration of appendages such as hair follicles through mediation of dynamic cell–cell interactions and regulation of wound microenvironment (Figure 2).

In the second research paper, extracts of *Flourensia cernua* (*F. cernua*) rich in bioactive polyphenols were encapsulated in collagen hydrogels in order to achieve their sustained release within skin wounds and thus increase their bioavailability and therapeutic efficacy. The effect of extracts’ concentration on the physicochemical and biological characteristics of the formed hydrogels were examined. It was found that an increase in the extract content accelerated the gelation time and enhanced the degree of crosslinking but decreased hydrogel stiffness as a result of the repulsion between the polymer chains and the extract molecules. However, independently of the extract content, the hydrogels exhibited sustained degradation under skin-mimicking conditions and hemocompatibility. Specifically, hydrogels with 14 wt% extract content were shown to increase the metabolic activity and proliferation of fibroblasts and monocytes, and to induce 90% wound contraction in 10 days in fibroblast cultures, indicating these hydrogels as bioactive scaffolds for wound healing.

Currently used chain-growth photopolymerization reactions for gelatin methacryloyl (GelMA) hydrogel crosslinking require high radical concentrations for initiation and long irradiation time, which could lead to cell cytotoxicity. Furthermore, difficulties in the modification of the hydrogel composition and functionalization have limited the application of covalently crosslinked gelatin hydrogels in 3D culture applications due to inability to tailor the microenvironment for specific types of cells. In the third research paper, a rapid photopolymerization reaction mechanism, based on thiolene click chemistry, has been proposed for the formation of gelatin hydrogels, where a thiol monomer (e.g., dithiothreitol (DTT)) reacts effectively with non-homopolymerizable alkenes (e.g., allyl glycidyl ether (AGE) coupled to gelatin) without requiring an increased radical concentration (Figure 3). Tuning of the hydrogel mechanical properties can be achieved via variations in the degree of gelatin functionalization with AGE, which depends on reaction conditions like allyl concentration, pH and time.

The fourth research paper deals with the development of biodegradable thermoresponsive hydrogels based on diblock and triblock copolymers of poly(lactic acid) (PLA) and poly(ethylene glycol) (PEG) (e.g., mPEG-PLA and PLA-PEG-PLA) for the targeted and controlled delivery of the iron-chelating drug deferoxamine (DFO) commonly used in thalassemia treatment. The copolymers were shown to exhibit thermoresponsive gelation at 37 °C, indicating their appropriateness for injectable drug delivery, where polymeric micelles, which act as drug carriers, undergo in situ gelation, thus enabling controlled drug release. DFO encapsulation efficiency was found to increase proportionally with the drug concentration, and DFO release was characterized by an initial burst release followed by a sustained release over seven days.

In the first review paper, the role of hyaluronic acid in the clinically challenging treatment of periodontitis, an inflammatory disease characterized by high microbial burden, inflammation and deterioration of periodontal tissues, is reviewed, placing an emphasis on the antimicrobial, immunomodulatory and regenerative potential of the polysaccharide. To date, due to its favorable behavior regarding the control of inflammation and the healing of soft tissues, HA has been applied as an adjunct in surgical and nonsurgical periodontal therapies and in minimally invasive regenerative strategies. In addition, HA-based hydrogels and hybrid systems encapsulating nanoparticles and/or bioactive agents have been developed and have been found to exhibit promising antibacterial and osteogenic potential in preclinical settings. However, the heterogeneity of the HA formulations studied so far, the inconsistency in the reporting of periodontal defect morphologies and the short follow-up periods have impeded the interpretation of generated data.

The final review paper focuses on the application of hydrogels in cardiac surgery, where their biocompatibility, high tunability of mechanical properties and increased water content permit the biomimicry of cardiac extracellular matrix and the support of cell viability and integration under dynamic conditions. More specifically, hydrogels offer encouraging solutions regarding regeneration of myocardium (e.g., injectable, patch-forming hydrogels) where they effectively reduce the infarct size, promote angiogenesis and preserve contractile function. In addition, concerning the engineering of heart valves, hydrogel matrices effectively contribute to scaffold construction due to their printability, mechanical resilience, compatibility with 3D fabrication and bio-responsiveness. Moreover, hydrogels aid in the prevention of pericardial adhesion and safe reoperation following cardiotomy. Additionally, hydrogel formulations facilitate topical delivery of small-molecule drugs and biomolecules, permitting sustained or stimulus-responsive drug release and reducing systemic toxicity. In this respect, despite shortcomings related to mechanical strength, translational scalability and immunocompatibility, hydrogels could be expected to assist cardiac surgery via novel, versatile and patient-specific therapies.

In conclusion, despite the significant progress made in the design and development of hydrogels for tissue repair, several critical barriers have hindered their translation to the clinic. Examples of these barriers include biocompatibility/biodegradability issues (e.g., accumulation of degradation products in the body resulting in inflammatory responses), inadequate matching of hydrogels with natural tissue interfaces, poor synchronization of hydrogel biodegradation with tissue formation, inadequate mechanical properties for load-bearing tissues such as bone and cartilage, lack of hydrogels with integrated antibacterial, pro-angiogenic and antioxidant properties, lack of data of preclinical evaluation in large animal models with closer physiological and anatomical resemblance to humans, scalability and regulatory issues, etc. [1,2]. The key to overcome the abovementioned barriers and achieve the development of translatable multifunctional hydrogels will be continuous technological progress based on multidisciplinary collaboration (i.e., integration of materials science with biology/immunology and clinical perspective).

## Figures and Tables

**Figure 1:**
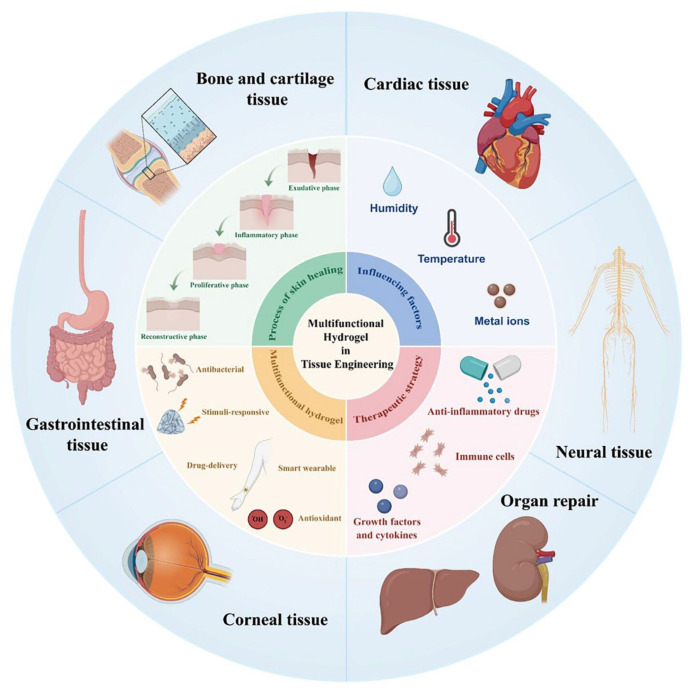
Schematic overview of multifunctional hydrogels in applications of tissue engineering and regenerative medicine [1].

**Figure 2:**
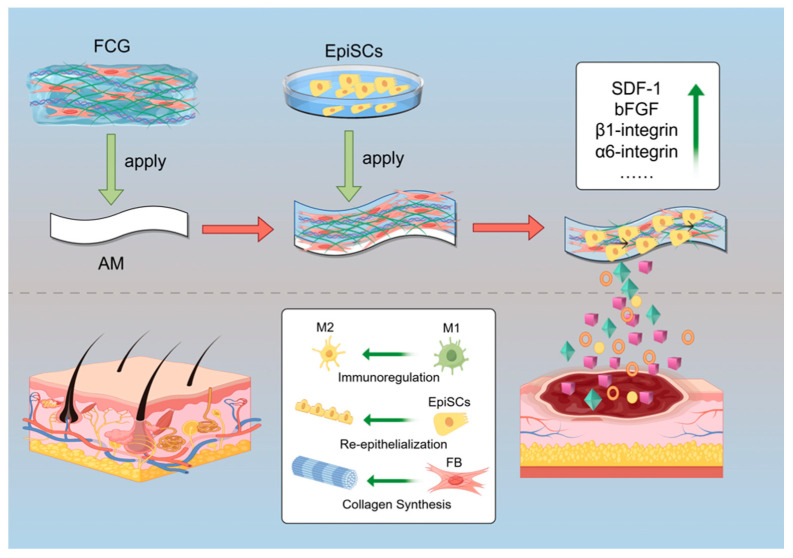
Schematic diagram of the fabrication process of the AM-FCG-EpiSCs. AM: amniotic membrane; FCG: fibroblast-laden collagen gel; EpiSCs: epidermal stem cells.

**Figure 3:**
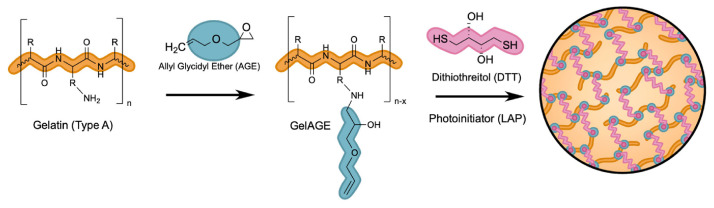
Functionalization of gelatin type A with AGE to form GelAGE hydrogel precursor, and subsequent UV crosslinking with DTT photoinitiated by LAP.

## Data Availability

Not applicable.
